# Using DIVAN to assess disease/trait-associated single nucleotide variants in genome-wide scale

**DOI:** 10.1186/s13104-017-2851-y

**Published:** 2017-10-30

**Authors:** Li Chen, Zhaohui S. Qin

**Affiliations:** 1 Department of Health Outcomes Research and Policy, Harrison School of Pharmacy, Auburn University, Auburn, AL 36849 USA; 2 Department of Biostatistics and Bioinformatics, Rollins School of Public Health, Emory University, Atlanta, GA 30322 USA; 3 Department of Biomedical Informatics, Emory University School of Medicine, Atlanta, GA 30322 USA

**Keywords:** Non-coding variants, D-score, DIVAN, Software

## Abstract

**Objective:**

The majority of sequence variants identified by Genome-wide association studies (GWASs) fall outside of the protein-coding regions. Unlike coding variants, it is challenging to connect these noncoding variants to the pathophysiology of complex diseases/traits due to the lack of functional annotations in the non-coding regions. To overcome this, by leveraging the rich collection of genomic and epigenomic profiles, we have developed DIVAN, or Disease/trait-specific Variant ANnotation, which enables the assignment of a measurement (D-score) for each base of the human genome in a disease/trait-specific manner. To facilitate the utilization of DIVAN, we pre-computed D-scores for every base of the human genome (hg19) for 45 different diseases/traits.

**Results:**

In this work, we present a detailed protocol on how to utilize DIVAN software toolkit to retrieve D-scores either by variant identifiers or by genomic regions for a disease/trait of interest. We also demonstrate the utilities of the D-scores using real data examples. We believe that the pre-computed D-scores for 45 diseases/traits is a useful resource to follow up on the discoveries made by GWASs, and the DIVAN software toolkit provides a convenient way to access this resource. DIVAN is freely available at https://sites.google.com/site/emorydivan/software.

**Electronic supplementary material:**

The online version of this article (10.1186/s13104-017-2851-y) contains supplementary material, which is available to authorized users.

## Introduction

Over the past decade, genome-wide association studies (GWASs) have successfully identified tens of thousands of single-nucleotide variants (SNVs) that show statistically significant association with thousands of diseases and traits. Databases have been developed to store those SNPs such as the Association Results Browser (ARB) (https://www.ncbi.nlm.nih.gov/projects/gapplus/sgap_plus.htm) and Genome-Wide Repository of Associations Between SNPs and Phenotypes (GRASP) [1].

An important finding from these studies is that most of the identified SNPs fall into the non-coding regions [2]. Unlike coding variants, how to gauge the functional impact of non-coding variants is a daunting challenge since they do not directly change the translated protein sequence. It is generally believed that non-coding variants interferes with the transcription factor (TF) binding and histone modification mechanisms of target genes [3], which subsequently affect the gene expression. Epigenomic data have thus been long recognized as an potential source of functional annotation for non-coding variants [4].

On the other hand, in recent years, large international consortia, such as ENCODE (the Encyclopedia of DNA Elements) [5] and the REMC (Roadmap Epigenomics Mapping Consortium) [6] have been commissioned to systematically conduct genome-wide profiling experiments including ChIP-seq [7], DNase-seq [8] and FAIRE-seq [9] across hundreds of cell lines/tissues. The publicly available epigenomic datasets offer a great resource to better understand the biology of the non-coding part of the genome [10].

Taking advantage of these valuable resources, multiple computational approaches have already been developed to annotate genetic variants using genome-wide profiling data including GWAVA [11], CADD [12], GenoCanyon [13], Eigen, EigenPC [14], DANN [15], fitCons [16], FATHMM [17], deltaSVM [18], dbNSFP [19], FunSeq 2 [20] and iCAGES [21]. A common feature of those methods is that they are disease/trait neutral, which means they only predict if a variant is deleterious or not, but not able to tell if a variant is likely to be associated with a particular disease/trait of interest. However, the latter is more of interest in the clinics.

To overcome the limitation, we recently developed a novel computational method named DIVAN (DIsease-specific Variant Annotation) [22], which is capable of gauging whether a mutation, no matter where it is located in the genome, is likely to be associated with a specific disease/trait. Like most of the existing methods, DIVAN offers a pre-computed functional score (referred to as the D-score) for every base of the entire human genome. The only difference is that these D-scores are disease/trait-specific. i.e., one set of scores for each disease/trait. For each disease, DIVAN model is trained using known GWAS variants with matching benign variants, and a set of informative features is selected from more than 1800 epigenomic profiles collected. We further develop a computational and memory efficient DIVAN software toolkit, which could be executed on a typical local computer.

In this work, for the sake of completeness, we first briefly describe the method and workflow of DIVAN, and then we present a detailed protocol on how to utilize the DIVAN software toolkit to obtain D-scores for a set of known variants, or a set of arbitrary genomic regions in a step-by-step manner.

## Main text

### Review of DIVAN

#### Construct positive and negative SNP sets

For each of the 45 diseases/traits studied, the set of disease/trait-associated SNVs (referred to as risk variants) identified by GWAS cataloged in ARB is treated as the positive set. To construct the corresponding negative set, we choose from all SNVs cataloged by the 1000 Genomes Project with minor allele frequency greater than 0.05 and, according to ARB, not associated with any known disease/trait (referred to as benign variants). We impose two criteria. The “distance to TSS-matched” criterion restricts that benign variants match those risk variants in terms of the distances to Transcription Start Site (TSS). The “region-matched criterion” requires that all benign variants located near (within 10 kb) of at least one risk variant. Given that there are way more benign variants than risk variants, the negative set is chosen to be 10 times the size of the positive set.

#### Collect epigenomic/genomic profiles from ENCODE and REMC

Epigenomics profiles including DNase-seq & FAIRE-seq characterizing open chromatin, and ChIP-Seq measuring histone modification, TF binding and RNA polymerase binding are collected from ENCODE and REMC. The genomic features mainly include repeated elements and conversation scores (GERP element [23] and phastCons scores [24]).

#### Annotate GWAS SNPs using epigenomic/genomic profiles

The entire genome is partitioned into consecutive windows of 200 bp. The read counts for these windows (adjusted for control data if available) are treated as the epigenomic features. In addition, we also annotate each window with presence or absence of repeat elements, GERP elements as genomic feature. We also use the phastCons scores for each window as another genomic feature. The result is a genome-wide annotation matrix with rows as 200 bp windows and columns as genomic and epigenomic features. Any variant is annotated with a full set of features by simply identifying the window that it falls into.

#### Build a disease/trait-specific feature-selection ensemble learning model

For each disease/trait, we first apply a feature selection step to select the informative features that better differentiate risk variants from benign variants. Specifically, for each feature, we apply a statistical test to measures the difference between positive and negative sets of variants. Cross-validation is applied to select an optimal threshold that decides which feature is deemed informative thus kept in the model. After the informative features are selected, an ensemble learning approach is used to build up multiple classifiers, each of which is assigned an equal number of risk and benign variants for training. Thus, given a variant/position, the prediction outcome is decided by the average of the votes from all classifiers, defined as the D-score, which could be interpreted as the probability of that variant/base being disease/trait-associated.

### Protocol

There are two ways to obtain D-scores for known variants: by variant identifiers or by genomic regions. For variant identifiers, DIVAN is capable of retrieving D-scores of known variants by variant identifiers or by genomic regions. We discuss the detailed step how to obtain the D-score of known variants by variant identifiers below. The detailed steps for how to retrieve D-scores of known variants by genomic regions and retrieve average D-scores for arbitrary genomic regions could be found in Additional file 1.

#### Retrieve D-scores of known variants by variant identifiers

First, download the set of pre-computed genome-wide base-level D-scores for the disease/trait of interest and variation database needed. For example, to retrieve D-scores for the Behcet Syndrome using the Ensembl variant identifiers, download files Emsembl.tar.gz, BehcetSyndrome.tar.gz and scoredistTSS.tar.gz and uncompress them into three folders “Ensembl”, “BehcetSyndrome” and scoredistTSS. Second, either run the R script “scoreDIVAN.cmd.R” in the command line or the R script “scoreDIVAN.console.R” inside an R console. Note that all the files, extracted folders and R scripts should be placed under the same directory before executing the command. In this example, use the command line

**Fig. 1:**
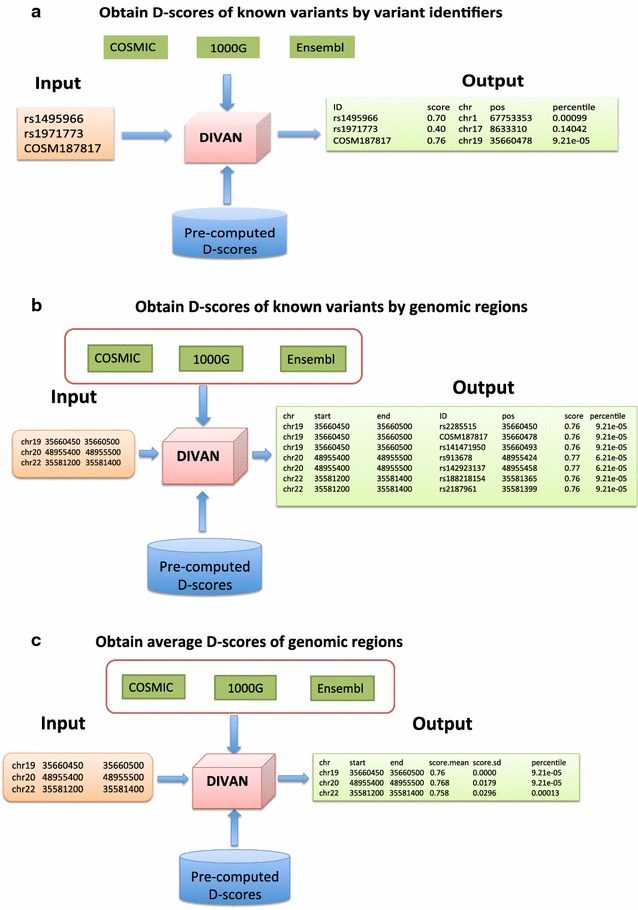
**a** Illustration of using DIVAN to obtain D-scores of known variants by variant identifiers. The input file contains a list of variant identifiers with each variant as one row. The output file contains tab-delimited columns representing variant identifier, D-score, chromosome, chromosome position and D-score percentile of each variant respectively. **b** Illustration of using DIVAN to obtain D-scores of known variants fall inside genomic regions of interest. The input file contains a list of genomic regions in the format of tab-delimited chromosome, start and end positions. The D-scores of known variants located within each genomic region are reported. The output file contains tab-delimited columns representing chromosome, start and end positions, variant identifier, position of variant and D-score with its corresponding percentile of each variant respectively. **c** Illustration of using DIVAN to obtain average D-scores of genomic regions of interest. The input file contains a list of genomic regions in the format of tab-delimited chromosome, start and end position. The mean and standard deviation of D-scores for all bases within each genomic region are calculated. The output file contains tab-delimited columns representing chromosome, start and end positions, mean of D-scores with the corresponding percentile and standard deviation of D-scores for each region respectively

*R* –*slave* –*args* –*no*-*save variant.txt BehcetSyndrome Ensembl scoredistTSS score.variant.txt* < *scoreDIVAN.cmd.R*

which takes input file “variant.txt” and generates output file “score.variant.txt”. The input file is formatted with each variant in one row. The output file contains the D-score with its corresponding percentile in the genome along with genomic position of each matched query variant. The illustration of the procedure is presented in Fig. 1a.

### Performance

DIVAN software works on a PC or laptop with less than 4 GB of memory. For a query of 125,713 regions (383 MB in total length), DIVAN only takes around 2 min. Moreover, the size of compressed file with pre-computed whole human genome base-level DIVAN score for one disease/trait is only around 100 MB. Therefore, DIVAN software is able to run on a regular PC or laptop. All the testing examples in the tutorial have been successfully performed on a MacBook laptop with a 1.7 GHz processor and 8 GB of memory.

### Real data examples

**Fig. 2:**
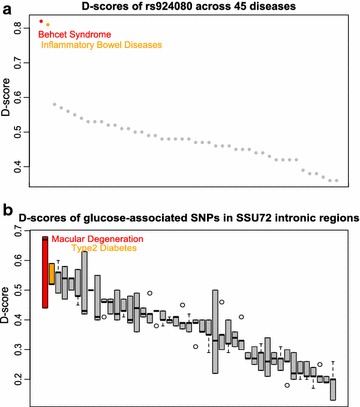
**a** D-score distribution for rs924080 for 45 diseases/traits. **b** D-score distribution of glucose-associated SNPs located in the SSU72 gene body for 45 diseases/traits

Behcet Syndrome (MIM 109650) is a rare disorder causing inflammation of the blood vessels and a genetically complex disease. Non-coding SNP rs924080 (chr1, 67760140) at the IL23R-IL12RB2 locus has been previously reported to be significantly associated with the Behcet Syndrome (*p* = 6.69 × 10^−9^, OR = 1.28) [25]. This SNP is also reported to be significantly associated with Inflammatory Bowel Diseases (MIM 612244) (p = 2.57 × 10^−6^) [26]. We obtain the D-scores of rs924080 across 45 diseases/traits studied (Fig. 2a). Clearly, The D-scores of rs924080 in Behcet Syndrome (0.82) and inflammatory bowel diseases (0.81) are significantly higher than the D-scores of other diseases. The finding is consistent with the two GWAS results.

It is also interesting to obtain the D-scores in genomic region of interest to investigate the functional connection between the genomic region and diseases/phenotypes. It is reported in dbGaP [27] that three SNPs located in the intronic region of gene SSU72 (chr1, 1477052- 1510261) have been identified by GWASs to be significantly associated with glucose level (rs3766178 (p = 3.26 × 10^−5^, chr1, 1542800), rs880051 (p = 1.89 × 10^−5^, chr1, 1558347), rs2296716 (p = 2.54 × 10^−5^, chr1, 1562444). The D-scores of the three SNPs across 45 diseases/traits are shown in Fig. 2b. It is not surprising to see that Type2 Diabetes (MIM 125853) ranks at the top as glucose in cells cannot respond to insulin correctly for Type2 Diabetes patients. It is also interesting to observe that D-scores in the three SNPs are quite high in Macular Degeneration. The metabolites of Glycolysis, which a critical pathway involves the metabolism of both glucose and lactate, has been reported to be abnormal in patients with Age-Related Macular Degeneration (MIM: 603075) [28]. Clearly, there exists a functional connection between the genomic region (glucose) and Type2 Diabetes as well as Macular Degeneration.

**Fig. 3:**
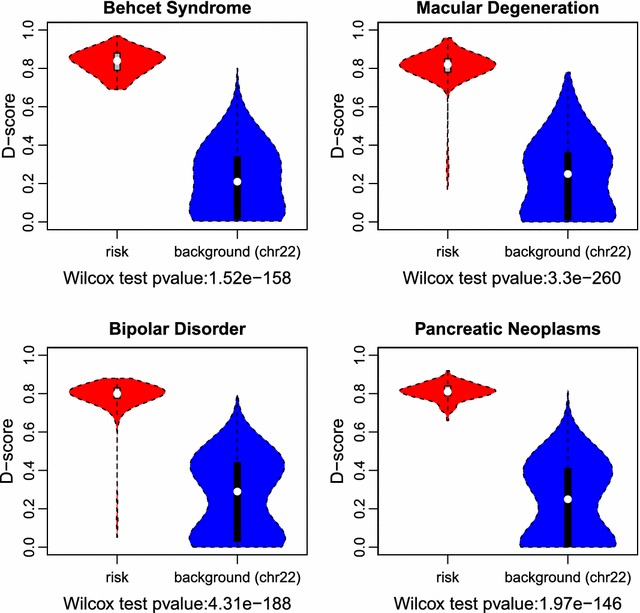
D-score distributions of the background (all bases in chr22) and risk variants associated with four diseases: Behcet Syndrome, Macular Degeneration, Bipolar Disorder and Pancreatic Neoplasms respectively

We further compare the distribution of D-scores of GWAS SNPs significantly associated with Behcet Syndrome, Macular Degeneration, Bipolar Disorder and Pancreatic Neoplasms in ARB and the background (taken to be all bases on chromosome 22) (Fig. 3). We perform the Wilcoxon Signed-Rank test between the D-scores of the risk variants and those in the background. As expected, we observe overall that the GWAS SNPs have significantly higher D-score than those in the background (p = 1.52 × 10^−158^ in Behcet Syndrome; p = 3.3. × 10^−260^ in Macular Degeneration; p = 4.31 × 10^−188^ in Bipolar Disorder; p = 1.97 × 10^−146^ in Pancreatic Neoplasms). Interestingly, we also find a few spots in the background that have higher D-scores than some of the GWAS SNPs. We hypothesized that those regions might harbor undiscovered novel risk variants those diseases.

## Limitations

Up to date, hundreds of diseases/traits have been studied in GWAS. In the future, we will pre-compute D-scores for more diseases/traits of interest besides the 45 diseases/traits already studied to make DIVAN software more comprehensive. Moreover, the calculation of current DIVAN score does not consider the order of GWAS p-values, which could be another important feature added into the training model. Other types of epigenomic features, including eQTL, DNA methylation, and pre-computed scores from GWAVA, CADD, and GenoCanyon could also be informative features to improve DIVAN further.

## Additional file

**Additional file 1.** The detailed protocol for using DIVAN in three cases: retrieve D-scores of known variants by genomic regions; retrieve average D-scores for arbitrary genomic regions; retrieve D-scores for multiple diseases/traits in batch.

## Abbreviations

GWAS: Genome-Wide Association Study
SNP: single nucleotide polymorphism
ARB: Association Results Browser
GRASP: Repository of Associations Between SNPs and Phenotypes
DIVAN: Disease-specific Variant ANnotation
ENCODE: encyclopedia of DNA elements
REMC: Roadmap Epigenomics Mapping Consortium
eQTL: expression quantitative trait loci
ChIP-seq: chromatin immunoprecipitation (ChIP) with massively parallel DNA sequencing
DNase-seq: DNase I hypersensitive site sequencing
FAIRE-seq: formaldehyde-assisted isolation of regulatory elements sequencing
GERP: genomic evolutionary rate profiling
