# Supplementary material for: Using DIVAN to assess disease/trait-associated single nucleotide variants in genome-wide scale
Source: BMC Res Notes. 2017 Oct 30;10:530. doi: 10.1186/s13104-017-2851-y (PMC5663107; doi:10.1186/s13104-017-2851-y)
Supplement: Supplementary file 1 — Additional file 1. The detailed protocol for using DIVAN in three cases: retrieve D-scores of known variants by genomic regions; retrieve average D-scores for arbitrary genomic regions; retrieve D-scores for multiple diseases/traits in batch. [file 13104_2017_2851_MOESM1_ESM.docx]

**Supplementary Materials**

**Using DIVAN to assess disease/trait-associated single nucleotide variants in genome-wide scale**

Li Chen^1*^, Zhaohui S. Qin^2,3*^

^1^Department of Health Outcomes Research and Policy, Harrison School of Pharmacy, Auburn University, Auburn, AL 36849, USA.

^2^Department of Biostatistics and Bioinformatics, Rollins School of Public Health, Emory University, Atlanta, GA 30322, USA.

^3^Department of Biomedical Informatics, Emory University School of Medicine, Atlanta, GA 30322, USA.

**Protocol**

**Retrieve D-scores of known variants by genomic regions**

First, download the set of pre-computed genome-wide base-level D-scores for the disease/trait of interest and variation database needed. For example, to retrieve D-scores for the Behcet Syndrome using the Ensembl variant identifiers in the genomic regions of interest, download files Emsembl.tar.gz, BehcetSyndrome.tar.gz and scoredistTSS.tar.gz, and uncompress both files into three folders “Ensembl”, “BehcetSyndrome” and “scoredistTSS”. Second, either run the R script “scoreDIVAN.cmd.R” in the command line or the R script “scoreDIVAN.console.R” inside an R console. Note that all the files, extracted folders and the R script should be placed under the same directory before executing the command. In this example, use the command line

*R --slave --args --no-save region.txt BehcetSyndrome Ensembl scoredistTSS score.region.txt < scoreDIVAN.cmd.R*

which takes input file “region.txt” and generates output file “score.region.txt”. The input file is formatted with each genomic region tab-delimited with chromosome, start position and end position in one row. The output file reports the D-score with its corresponding percentile in the genome of known variants in the Ensembl database within each genomic region of interest. The illustration of the procedure is presented in Figure 1B.

**Retrieve average D-scores for arbitrary genomic regions**

If the variant of interest is novel, or the user is interested in a particular genomic locus or set of loci, users can use the genomic regions to query. First, download the set of pre-computed genome-wide base-level D-scores for the disease/trait of interest. For example, to retrieve D-scores for the Behcet Syndrome in some genomic regions, download files BehcetSyndrome.tar.gz and scoredistTSS.tar.gz, and uncompress them to obtain two folders “BehcetSyndrome” and “scoredistTSS.tar.gz”. Second, either run the R script “scoreDIVAN.cmd.genome.R” in the command line or the R script “scoreDIVAN.console.genome.R” inside an R console. Note that all the files, extracted folders and the R script should be placed under the same directory before executing the command. In this example, the command line

*R --slave --args --no-save region.txt BehcetSyndrome scoredistTSS score.genome.txt < scoreDIVAN.cmd.genome.R*

which takes input file “region.txt” and generates output file “score.genome.txt”. The input file is formatted with a genomic region tab-delimited with chromosome, start position and end position in one row. The output file reports an average D-score with its percentile along with the standard deviation of the D-scores for all bases within each genomic region. The illustration of the procedure is presented in Figure 1C.

**Retrieve D-scores for multiple diseases/traits in batch**

DIVAN software is now capable of retrieving D-scores of multiple diseases/traits for a set of variants or regions simultaneously. The command line is similar to aforementioned in each scenario and the only difference is to replace the folder corresponding to one disease/trait that contains the pre-computed D-scores to a file that lists all folders corresponding to multiple diseases/traits. More details can be found in the online tutorial (https://sites.google.com/site/emorydivan/tutorial).
